# Approximate Optimization Study of Light Curing Waterborne Polyurethane Materials for the Construction of 3D Printed Cytocompatible Cartilage Scaffolds

**DOI:** 10.3390/ma14226804

**Published:** 2021-11-11

**Authors:** Yi-Wen Chen, Ming-You Shie, Wen-Ching Chang, Yu-Fang Shen

**Affiliations:** 1 x-Dimension Center for Medical Research and Translation, China Medical University Hospital, Taichung City 40447, Taiwan; evinchen@gmail.com (Y.-W.C.); eviltacasi@gmail.com (M.-Y.S.); jayla11218@gmail.com (W.-C.C.); 2 Graduate Institute of Biomedical Sciences, China Medical University, Taichung City 40447, Taiwan; 3 School of Dentistry, China Medical University, Taichung City 40447, Taiwan; 4 Department of Bioinformatics and Medical Engineering, Asia University, Taichung City 41354, Taiwan; 5 High Performance Materials Institute for xD Printing, Asia University, Taichung City 41354, Taiwan

**Keywords:** cartilage scaffold, light curing waterborne polyurethane, digital light processing, design of experiments

## Abstract

Articular cartilage, which is a white transparent tissue with 1–2 mm thickness, is located in the interface between the two hard bones. The main functions of articular cartilage are stress transmission, absorption, and friction reduction. The cartilage cannot be repaired and regenerated once it has been damaged, and it needs to be replaced by artificial joints. Many approaches, such as artificial joint replacement, hyaluronic acid injection, microfracture surgery and cartilage tissue engineering have been applied in clinical treatment. Basically, some of these approaches are foreign material implantation for joint replacement to reach the goal of pain reduction and mechanism support. This study demonstrated another frontier in the research of cartilage reconstruction by applying regeneration medicine additive manufacturing (3D Printing) and stem cell technology. Light curing materials have been modified and tested to be printable and cytocompatible for stem cells in this research. Design of experiments (DOE) is adapted in this investigation to search for the optimal manufacturing parameter for biocompatible scaffold fabrication and stem cell attachment and growth. Based on the results, an optimal working process of biocompatible and printable scaffolds for cartilage regeneration is reported. We expect this study will facilitate the development of cartilage tissue engineering.

## 1. Introduction

Articular cartilage disease is an important issue that seriously influences the quality of life of millions of people every year. Osteoarthritis (OA) is the most common form of degenerative joint disease, and the quality of life of people with OA is severely affected [1]. It affects more than 40 million people every year [2], and 70% of the population over 65 years of age suffers from it [3]. Neither the use of drugs nor surgical treatment can fully replace the structure and function of cartilage. However, recent tissue engineering technology provides the possibility to improve and develop the regeneration and restoration of defected cartilage tissue.

The main function of articular cartilage is to withstand friction, provide compressive resistance and protect the subchondral bone. Unlike most other tissues, cartilage does not have blood vessels and has low metabolic activity. This avascular structure means the lack of nutrients and precursor cells, severely limiting their ability to self-regenerate [4]. Therefore, the ability to repair cartilage damage is limited. In addition, minor defects in articular cartilage are often difficult to detect. When pain occurs, it is often related to an injury to the subchondral bone [5]. Since the mechanical wear rate of articular cartilage is much higher than that of cartilage, chronic and progressive degenerative joint diseases such as osteoarthritis (OA) and rheumatoid arthritis (RA) are inevitable, and easily lead to chronic pain and disability. Nevertheless, the cartilage tissue currently prepared by tissue engineering still differs from the natural cartilage in its structural composition and its physical and mechanical properties. Treatments include articular surface ablation, arthroscopic cartilage surgery, mosaicplasty, autologous chondrocyte transplantation, and allogeneic cartilage transplantation. Articular surface ablation can cause thermal necrosis of cells. Arthroscopic cartilage surgery is only suitable for small areas of cartilage damage. Mosaicplasty removes the cylindrical bone and cartilage from the smaller part of the knee and then inlays it in the injured position but the availability of the donor site and the quality of the graft is a limitation of this technique [5]. Autologous chondrocyte transplantation is only effective in early cartilage lesions, but if the operation is delayed, the effect is not satisfactory, and the cartilage taken out at the transplant site may be damaged due to long-term abnormal weight bearing [6,7,8]. Allogeneic soft bone grafts have a risk of causing the body to be immune to reactions and infections. Therefore, the reconstruction of the articular cartilage defects remains unsatisfactory and perfect, and there is still need for further development to solve the problem [9]. Traditional scaffold manufacturing techniques include solvent–casting, particulate-leaching [10,11], phase separation [12], freeze drying and electrospinning [13]. However, there are still many problems and challenges in the manufacture of ideal biological scaffolds by traditional scaffold manufacturing techniques. The main reason is that the traditional manufacturing technology can’t quickly and freely design the internal and external structures of the scaffolds. Nevertheless, scaffolds made by three-dimensional printing technology can have complex internal three-dimensional porous structures for cell attachment, proliferation and nutrient transport. More importantly, combined with medical imaging technology and computer three-dimensional drawing software, the shape of the customized scaffold can be prepared to match the cartilage damage area [14,15,16], which helps with the development of cartilage tissue engineering. With the development of three-dimensional (3D) printing technology, this technology has been recognized as having advantages including high precision, rapid manufacturing and customized production, and has great application potential in the fields of regenerative medicine and tissue engineering. Previous studies have separately reported the method of manufacturing cartilage scaffolds with 3D printing technologies such as the new multi-head deposition system [17] and liquid-frozen deposition manufacturing (LFDM) [18]. These research results also prove that 3D printing technology has significant potential in the development of cartilage tissue engineering. Only the mechanical properties and printing accuracy still need to be improved.

Among the previous research technologies of 3D printed cartilage scaffolds, there has been fused deposition manufacturing (FDM) [19], selective laser sintering (SLS) [20,21], stereolithography (SLA) [22], digital light processing (DLP) [23] and 3D bioprinting [17], etc. Both synthetic and natural materials are able to be used in 3D printing to create cartilage tissue scaffolds. Although FDM technology has the advantage of lower manufacturing cost, its accuracy is lower. In addition, SLS technology will generate a relatively large amount of waste. SLA and DLP have better printing accuracy [24], but most of the materials use organic solvents, which affects biocompatibility [25]. Therefore, non-toxic light-cured materials that can be used for SLA and DLP have become the main focus in recent years [26,27,28].

In 2007, Bens’ research team developed a biocompatible photocurable material (FlexSL^®^) that can be used in medical applications for short-term contact with bones, teeth or tissues with a cell survival rate of 87% [29]. In the following year, Sharifi and other scholars reported that the work pieces printed on different materials developed by them could have different Young’s modulus, and the Young’s modulus ranged from 0.4 to 8000 MPa, indicating that SLA can produce complex work pieces with different mechanical properties [30]. In 2009, Melchels et al. began to develop composite materials containing photosensitive materials and non-photosensitive materials [27], and in 2010 the produced biological scaffolds and confirmed the use of biological scaffolds made with SLA technology that had good permeability [31]. In 2008, Han et al. mixed a low molecular weight PEGDA polymer with an inert liquid to make a scaffold and observed the attachment of cells to the scaffold. However, scaffolds made from this PEGDA material are not degradable [32]. In 2009, Choi et al. produced three-dimensional scaffolds from biodegradable materials, but there were uncured material residues inside the scaffolds and no biocompatibility tests were performed in this research [24]. In 2010, Tanaka and other scholars compared traditional manufacturing technology with FDM technology in additive manufacturing technology to measure the size and porosity of biological scaffolds and perform indentation force and Young’s modulus tests. The physical properties of the fabricated scaffolds were also compared to natural cartilage and animal experiments were performed to assess the benefits of cartilage repair [33]. In 2017, our research group developed cytocompatible water-based light-cured polyurethane related materials for DLP’s 3D printing technology [34]. The previous results showed the materials were printable and can be printed as a customized scaffold, and the printed scaffolds can help cells have good differentiation potential [34]. The three-dimensional printing technology can produce cartilage scaffolds with complex internal three-dimensional porous structures and different layers of different structures, providing cell attachment, proliferation, and nutrient transport. In addition, the development of hydrogel materials with encapsulated cells enhances the potential of stem cells to differentiate into chondrocytes and promotes cartilage tissue maturation. Furthermore, this study, in conjunction with an extrusion-type printing system, developed a new process to prepare a customized hybrid multi-layered scaffold.

This study used the Design of experiments (DOE) model to develop biomedical materials that can be applied to DLP technology, and this material can meet the needs of the manufacturing of cartilage scaffolds, combined with biological scaffold design and machine process parameters. The optimization of the cartilage scaffold process is achieved, making it suitable for cartilage tissue engineering. Previous studies have focused on how to develop photocurable materials with biomedical material properties, but few studies have focused on DLP technology machine parameters and bio-scaffold design. This study optimized the material development, biological scaffold design, and process parameters. This study analyzes and discusses the response variables of different factors and provides the best parameters for the manufacture of biomimetic cartilage scaffolds. Therefore, this study will contribute to the development of cartilage tissue engineering and can be applied to knee joints in the future, thus treating cartilage damage and achieving the goal of customizing cartilage scaffolds.

## 2. Materials and Methods

### 2.1. Experimental Design

In order to simplify and improve the characteristics of the experiments, this study uses experimental design and statistical evaluation methods to evaluate and identify the most important parameters and possible interactions of these parameters [35]. Resolution statistical design was applied to the polyurethanes/thermoplastic polyurethanes (PU/TPU, Alberdingk Boley, Krefeld, Germany) materials formulation containing 2-Hydroxylethyl methacrylate (HEMA, Sigma-Aldrich, St. Louis, MO, USA) and the designed scaffold structure to consider the parameters in two different extreme levels (Table 1). The parameters contain several factors: experimental factor A is the ratio of water-based light-cured PU in the PU/TPU mixture (%); factor B is the HEMA addition amount; factor C is curing time per layer (s); factor D is pore size of the scaffold (mm); and factor E is porosity of the scaffold (%). The significant influence of these parameter independent variables (Table 1) on the dependent variable was examined, including diametral tensile strength (DTS) (response 1), Young’s modulus (response 2), cell adhesion amount (response 3), and cell viability (response 4), using Design-Expert^®^ (Stat-Ease, Minneapolis, MN, USA) with Experimental combination of 2^5−2^ partial factor experimental design. The setting of DTS and Young’s modulus response values is based on previous research [36]. The range of the set response variable is shown in Table 2. The design produced eight experiments (Table 3), and each experiment performed at least three independent experiments. Table 3 shows the results of eight experiments after two rounds.

### 2.2. The Preparation of Water-Based Light-Cured PU/TPU Composites

0–50% water-based thermoplastic polyurethanes (TPU; U 2101, Alberdingk Boley, Krefeld, Germany) was added to water-based light-cured polyurethanes (PU; LUX 260, Alberdingk Boley, Krefeld, Germany). The solid content of U 2101 and LUX 260 are 50% and 40%, respectively. After mixing, the hybrid materials were heated at 180 °C and stirred at high speed for 1 h to remove the water. Amounts of 1.5% 2,4,6-trimethylbenzoyl-diphenyl-phosphineoxide (TPO, Ciba, Basel, Switzerland) photoininitiators were dissolved in 2-Hydroxylethyl methacrylate (HEMA, Sigma-Aldrich, St. Louis, MO, USA). The weight of HEMA is 0.1–0.3 times the weight of the hybrid material after removing water. They are added to the water-based light-cured PU or PU/TPU hybrid materials to mix at 60 °C for 3D printing.

### 2.3. Graft Fabrication

**Figure 1:**
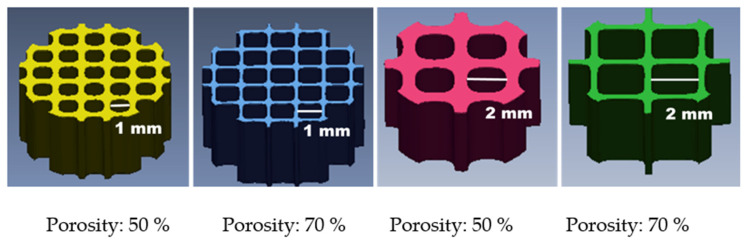
The design of cartilage scaffold. A cylinder with a radius of 3 mm and a height of 3 mm, and input the monomer size and porosity according to the software requirements.

The overall size of the scaffolds was fixed to a cylinder with a radius of 3 mm and a height of 3 mm. All scaffolds (Figure 1) were designed through SolidWorks (Dassault Systemes SolidWorks Corp., Waltham, MA, USA) and the 3-matic software (Materialise, NV, Leuven, Belgium) was used to choose and design the monomer size (side length: 1 or 2 mm), monomer shape (rounded square) and porosity (50 or 70%). They were fabricated by a MiiCraft high resolution DLP 3D printer (Young Optics Inc., Hsinchu, Taiwan) with blue light (405 nm) and the digital stereolithography 3D printing technology. Their manufacturing parameters are 50 μm per layer of at 7 to 25 s exposure. The uncured materials were washed away, and the objects were post-cured under ultraviolet light to obtain fully cured objects. The cured objects were washed again for cell adhesion and viability tests.

### 2.4. Mechanical Testing

The mechanical properties of the samples were examined using an EZ-Test machine (Shimadzu, Kyoto, Japan) with a 500 N load cell at a loading rate of 40 mm/s. The failure load L was used in conjunction with the diameter D and height h of the specimens to calculate DTS according to the relationship DTS = 2 L/πDh. The Young’s modulus (E = σ/ε, where σ and ε denote the stress and strain of the sample, respectively) was calculated from the linear region in a stress-strain curve. The samples of each condition were measured three times and the average value was recorded.

### 2.5. Cell Adhesion Test

The scaffolds were placed in a 24-well culture plate and sterilized for 1 h under ultraviolet light. Approximately 10^5^ human Wharton’s Jelly mesenchymal stem cells (WJMSCs, Bioresource Collection and Research Center, Hsin-Chu, Taiwan) were seeded and cultured for 3 h, and then were transferred to a new 24-well culture plate. The WJMSCs (passages 6–10) were maintained in DMEM medium (Gibco) supplemented with 10% fetal bovine serum (Gibco), penicillin (50 U/mL, Life Technology, Thermo Fisher Scientific Inc., Waltham, MA, USA), and streptomycin (50 µg/mL, Life Technology, Thermo Fisher Scientific Inc., Waltham, MA, USA). The scaffolds with cells were washed with phosphate-buffered saline (PBS), and 1 mL of trypsin per well was added to suspend the cells. The total number of the adhesion cells per scaffold was counted by a hemocytometer.

### 2.6. Cell Viability Test

The experiment was divided into an experimental group and a control group. There were cells and scaffolds in the experimental group, and only cells were placed in the control group. The scaffolds were placed in a 24-well culture plate and sterilized for 1 h under ultraviolet light. Approximately 10^5^ WJMSCs were seeded and cultured for 24 h. The WJMSCs (passages 6–10) were maintained in DMEM medium (Gibco, Thermo Fisher Scientific Inc., Waltham, MA, USA) supplemented with 10% fetal bovine serum (Gibco, Thermo Fisher Scientific Inc., Waltham, MA, USA), penicillin (50 U/mL, Life Technology, Thermo Fisher Scientific Inc., Waltham, MA, USA), and streptomycin (50 µg/mL, Life Technology). After 24 h of culture, the cell viability assay was performed by the PrestoBlue (Invitrogen, Grand Island, NY, USA) assay. After adding the PrestoBlue reagent for 1 h, the absorbance at wavelengths of 570 and 600 nm (reference wavelength) were measured with a multi-well spectrophotometer Infinite M200 PRO (Hitachi, Tokyo, Japan). The data of the control group was set to a survival rate of 100%, and the relative cell survival rate of the experimental group was calculated.

### 2.7. Statistics

The experimental results were confirmed by at least three independent experiments and regression analysis, and the data were all expressed as mean ± standard error (SE). The statistical significance of the process parameters was verified by the analysis of variance (ANOVA) method and analyzed with a confidence level of 9X% (α = 0.0 (10 − X)).

## 3. Results and Discussion

**Figure 2:**
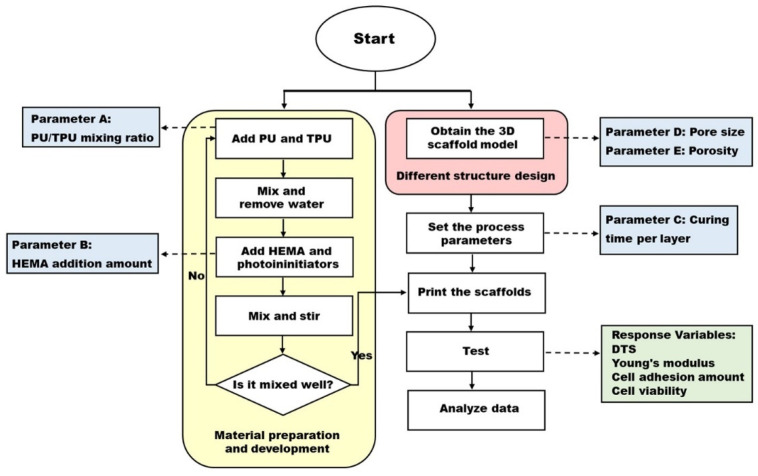
Flow chart of the experiments.

The development of cartilage tissue engineering has a history of more than 20 years [37], but the development of cartilage scaffold materials and manufacturing is still an important issue. Even though we have developed cytocompatible water-based light-cured polyurethane related materials and made it possible to manufacture cartilage scaffolds by DLP’s 3D printing technology in our previous research [34], we did not discuss the interactions related to the material ratio, the machine parameters of the DLP technology and the design of the scaffolds. Therefore, DOE was used in this study to optimize the material formulation, scaffold design and process parameters, and 2^5−2^ partial factorial experimental design was selected to achieve the maximum benefit with the least resources. The experimental flowchart is shown in Figure 2. Furthermore, the statistical analysis was applied in this study to obtain a significant model for further research and development in the future.

### 3.1. Diametral Tensile Strength

DTS is one of the mechanical properties of cartilage scaffolds. The ANOVA results of DTS are shown in Table 4. The model is significant with a coefficient of determination (R^2^) value of 95.45% and an adjusted coefficient of determination (adjusted R^2^) value of 92.41%. The results indicate that the factors (A), (C), (D) and the interaction between factors (B) and (E) are significant factors (*p* < 0.05). Since the DTS value of water-based light-cured PU is high and the DTS value of water-based TPU is low [34], the DTS value of the scaffold is affected as the mixing ratio of PU/TPU is different. This explains why the significant factor calculated by statistical analysis is (A). Furthermore, the curing time may affect the curing reaction, thereby affecting the DTS [38]. The ANOVA results also pointed out that (C) is a significant factor of DTS. 

**Figure 3:**
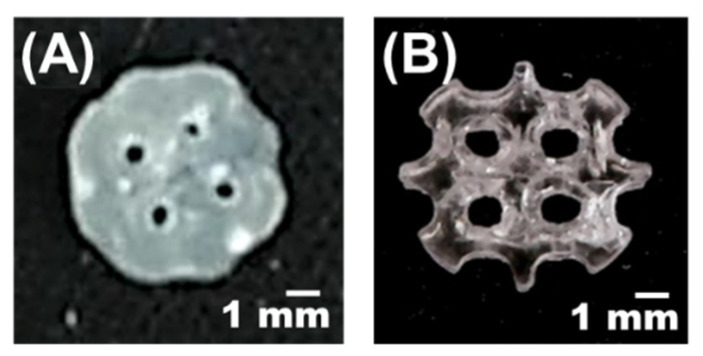
Comparison of the different HEMA addition amount with the same porosity and pore size of the scaffolds. The weight of HEMA is (**A**) 0.1 and (**B**) 0.3 times the weight of the PU/TPU mixture.

The left 1 and the left 3 of Figure 1 are scaffolds with the same porosity and different pore sizes. It can be seen from Figure 1 that the size of the pores will affect the structure of the scaffold. The difference of the scaffold structure causes the difference in DTS. In this study, the pore size (D) was determined to be a significant factor of DTS through ANOVA analysis. Figure 3 shows the printed scaffolds with the different HEMA addition amounts. HEMA was used as a thinner in this study to adjust the viscosity of printed materials. When the HEMA addition amount is low, the material is more viscous, which affects printing accuracy. It causes the diameter of the printed scaffold to be larger than that of the original design structure, and the pore size becomes smaller. Furthermore, the porosity of the designed scaffold may affect the required printing accuracy. Therefore, the interaction between factors (B) and (E) was also determined to affect the DTS of the scaffolds by the ANOVA analysis. The equation for DTS as obtained from the experimental data is shown by the following equation with 95.45% coefficient of determination (R^2^).

DTS = 3.42 + 2.19 × (A) − 0.56 × (B) + 1.79 × (C) − 1.41 × (D) + 0.56 × (E) − 1.32 × (B) × (E)(1)

### 3.2. Young’s Modulus 

**Figure 4:**
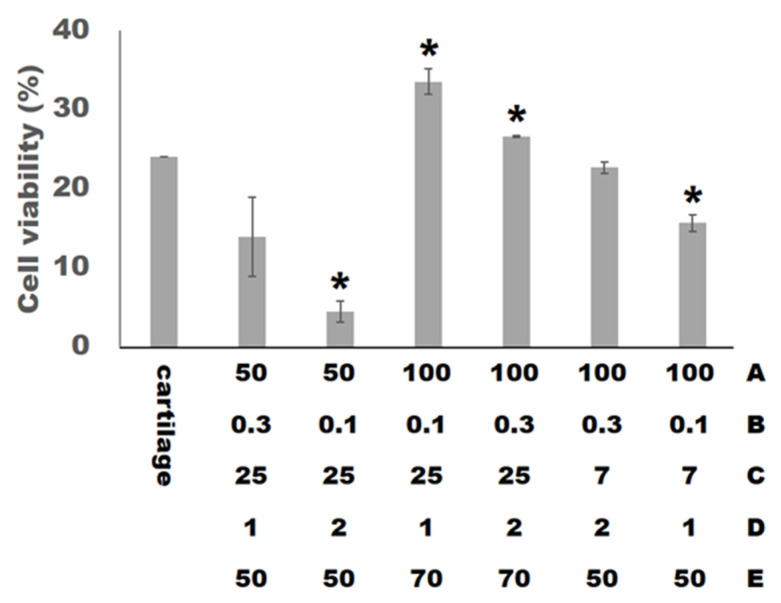
The Young’s modulus of the scaffolds compared with natural articular cartilage. The asterisk (*) indicates *p* < 0.05 versus cartilage.

Scaffolds in cartilage tissue engineering need to imitate natural articular cartilage so that they can have similar mechanical properties, such as with Young’s modulus. The result of the Young’s modulus is shown in Figure 4. Table 5 shows the ANOVA results of Young’s modulus. The model is significant, with a coefficient of determination (R^2^) value of 97.40% and an adjusted coefficient of determination (adjusted R^2^) value of 96.10%. The factors (A), (C) and the interaction between factors (B) and (E) were identified as significant factors in this analysis (*p* < 0.05).

Since both the Young’s modulus and the DTS are mechanical properties of the scaffolds, we speculate that the factors that affect the value of the Young’s modulus are the same as the DTS. In the ANOVA analysis, it is indeed shown that the significant factors of the Young’s modulus and the DTS are similar, and the only difference is that the significant factor of Young’s modulus does not contain a factor (D). The equation for Young’s modulus is shown in Equation (2), and R^2^ is 97.40%.

Young’s modulus = 14.59 + 10.01 × (A) + 1.18 × (B) + 5.01 × (C) + 0.43 × (E) − 2.92 × (B) × (E)(2)

### 3.3. Cell Adhesion Amount

As a scaffold for cartilage tissue engineering, we expect that the amount of cells attached to the scaffold can be as large as possible, so we set the range of its response variable to maximize. The ANOVA results of the cell adhesion amount are shown in Table 6. The model is significant, with a coefficient of determination (R2) value of 97.40% and adjusted coefficient of determination (adjusted R2) value of 96.10%. The results of Table 6 pointed out the factors (A), (C), (D), (E) and the interaction between factors (B) and (C) are the significant ones (*p* < 0.05) with regard to the cell adhesion amount on the scaffolds.

The cell adhesion amount on the scaffold is affected by the surface area of the scaffold. The larger the surface area of the scaffold, the greater the number of cells that can be attached. Both (D) and (E) are directly related to the surface area of the scaffold. Besides, the previous discussion showed that (C) can cause the diameter of the scaffold to become larger (Figure 3) and indirectly affect the surface area. This analysis confirms that the factors (D), (E) and (C) are significant factors affecting the amount of cell adhesion.

Although the physical properties of the scaffold can be adjusted by adding water-based TPU, we found that the printing accuracy will decrease accordingly. This is because TPU does not have photocurable functional groups. Therefore, the photocuring reaction will be affected with the addition of the TPU. Due to the decrease in printing accuracy, the diameter of the printed scaffold becomes larger, the pores become smaller, and the surface area increases. In this ANOVA analysis, factor (A) was determined to be a significant factor. In addition, the interaction between factors (B) and (C) can also influence the surface area of the printed scaffold. When setting a higher curing time and adding a smaller amount of HEMA, the surface area of the printed scaffold will increase significantly. The ANOVA analysis also pointed out the interaction between the factors (B) and (C) as a significant factor of the cell adhesion amount. In this experiment, in order to make the model significant and to determine the significant factor, the formula $y^{\prime }=\left(y+k\right)$ is used for conversion, where y is the measured value. The equation for the cell adhesion amount is shown in Equation (3), and R^2^ is 93.66%.

(Cell adhesion amount)^2^ = 4.55 + 2.51 × (A) − 0.48 × (B) + 1.26 × (C) − 1.92 × (D) − 0.78 × (E) + 0.92 × (B) × (C) (3)

### 3.4. Cell Viability

**Figure 5:**
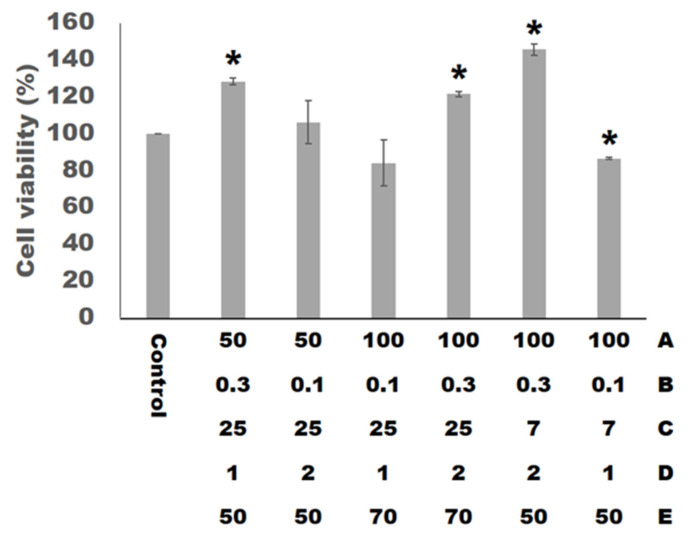
The cell viability of hWJMSCs cultured on the scaffolds for 24 h. The asterisk (*) indicates *p* < 0.05 versus control.

The biomedical materials used for cartilage tissue engineering must be biocompatible. According to ISO 10993-5, cell viability percentages below 40% are considered strong cytotoxicity; 40–60% moderate; 60–80% weak and over 80% are non-cytotoxic respectively [39]. Figure 5 shows the result of the cell viability with the normal tissue culture plates (Control). In this experiment, we set the range of the cell viability response variable to maximize. The ANOVA analysis of the cell viability is shown in Table 7. The model is significant, with a coefficient of determination (R^2^) value of 99.29% and adjusted coefficient of determination (adjusted R^2^) value of 98.81%. All factors and the interaction between factors (B) and (E) are the significant factors (*p* < 0.05) of the cell viability.

The photoinitiators in the materials are slightly toxic. If the light curing reaction is not complete, the toxicity will increase. The setting of curing time (C) will affect the completeness of the light curing reaction, and the factor (C) was judged as a significant factor affecting cell viability in this experimental ANOVA analysis. In addition, cell viability is related to the total volume of the material. As the total volume of the material is larger, the amount of photoinitiator is higher, and it is less conducive to cell growth. The factors (D) and (E) are related to the total volume of the scaffolds and were also identified as significant factors through the ANOVA analysis. Furthermore, all the factors and the interaction between factors (B) and (E) will affect the appearance of the printed scaffolds, thereby affecting the total volume of the scaffolds, and are considered to be significant factors by the ANOVA analysis. By the way, in addition to the influence of the total volume of the scaffold, previous studies have pointed out that porosity and pore size will affect cell viability. When the porosity of the scaffold is higher, the flow of gas and nutrients is smoother, and the cell growth is better. The previous studies have shown that the pore size of the scaffold is bigger than 300 μm to facilitate cell growth, and the pore size set in this study is all greater than 300 μm. The equation for the cell viability as obtained from the experimental data is shown by the following equation with 99.29% coefficient of determination (R^2^).

4Cell viability = 83.96 + 25.40 × (A) + 14.82 × (B) + 25.96 × (C) + 9.28 × (D) − 32.60 × (E) − 5.48 × (B) × (E)(4)

### 3.5. The Prediction and Verification of Optimal Factor-Level Combination 

**Figure 6:**
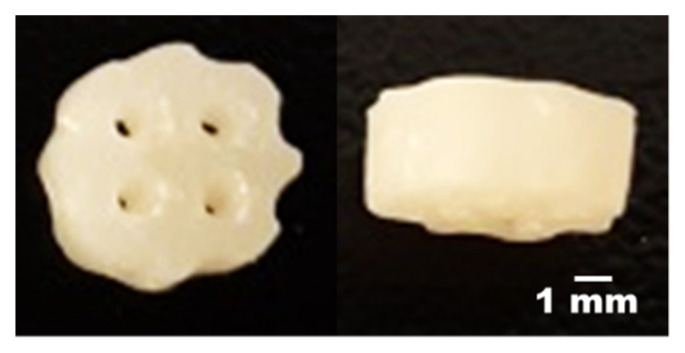
The images of the printed scaffold with the optimization parameter.

According to the statistical analysis results, the optimal factor-level combination can be obtained (Table 8). The verification result of the combination 4 is shown in Table 9 and Figure 6. The optimal factor-level combination is the mixing ratio (A) of 76.37%, the HEMA amount (B) of 0.2998 times, the curing time (C) of 23.08 s per layer, the pore size (D) of 2 mm and the porosity (E) of 60%. Through the confirmation experiment, the actual response value of DTS measured is 3.05 MPa, and the deviation is 4.59%; the Young’s modulus is 22.37 MPa, and the deviation is 10.59%; the amount of cell attachment is 2.75 × 10^4^, and the deviation is 27.90%; the cell viability is 106.89%, and the deviation is −17.82%. The results show that optimized conditions can improve the cell adhesion rate, biocompatibility and similarity of mechanical properties [40].

Previous studies have shown that the DTS range of human articular cartilage is between 0.6 and 4.5 MPa [41], and the Young’s modulus is between 20 and 30 MPa [42]. Although the experimental response variables of DTS and Young’s modulus have deviation, their experimental values are in the range similar to that of natural cartilage. The experimental value of the cell adhesion amount is better than the predicted value. In addition, ISO10993 stipulates that materials with cell viability greater than 80% are regarded as non-toxic [39]. Although the experimental value of cell viability is less than the predicted value, its value is much greater than 80%. Therefore, the scaffold made by the optimal factor-level combination is not toxic. 

## 4. Conclusions

This study used the DOE to develop water-based polyurethane hybrid materials that can be applied to DLP technology, and these materials have printability, mechanical properties similar to cartilage [41,42], have previously been reported to have the potential of cartilage differentiation [34], and can be used as a candidate for cartilage scaffolds [34]. Combined with scaffold design and machine process parameters, the optimization (Parameter A: 76.4; B: 0.3; C: 23; D: 2; E: 60) of cartilage scaffold manufacturing is realized, and it shows a good cell adhesion amount and viability and it can be used for cartilage tissue engineering. In addition, the customized porous scaffolds which are similar to the shape of the cartilage defect of the recipient site can be designed through reverse engineering. The scaffolds can be printed via DLP technology for the precise procedures of cartilage tissue reconstruction in the future [43].

## Figures and Tables

**Table 1:** Experimental factors and levels.

Experimental Factor	A	B	C	D	E
Low level	50	0.1	7	1	50
High Level	100	0.3	25	2	70

**Table 2:** Range of response variables.

Response Variables	Range
DTS (MPa)	0.6–4.5
Young’s modulus (MPa)	20–30
Cell adhesion amount (pc) Cell viability (%)	Max Max

**Table 3:** Experimental factors (columns 2–6) and response variables (columns 7–10) of the 2^5−2^ fractional factorial screening design (#1–#16).

Run #	A	B	C	D	E	DTS (MPa)	Young’s Modulus (MPa)	Cell Adhesion Amount (pc)	Cell Viability (%)
1	50	0.3	25	1	50	5.05	17.39	2.78 × 10^4^	126.82
2	50	0.3	25	1	50	3.27	10.34	2.5 × 10^4^	129.6
3	50	0.1	25	2	50	0.72	3.52	1.1 × 10^4^	97.74
4	50	0.1	25	2	50	0.84	5.39	1.1 × 10^4^	114.35
5	100	0.1	25	1	70	11.03	34.64	3.05 × 10^4^	92.93
6	100	0.1	25	1	70	12.42	32.41	3.13 × 10^4^	75.13
7	100	0.3	25	2	70	4.06	26.64	2.2 × 10^4^	120.43
8	100	0.3	25	2	70	4.35	26.48	2.5 × 10^4^	122.38
9	50	0.3	7	1	70	0	0	0	0
10	50	0.3	7	1	70	0	0	0	0
11	100	0.3	7	2	50	3.00	22.15	1.65 × 10^4^	143.35
12	100	0.3	7	2	50	3.16	23.12	2.2 × 10^4^	147.7
13	50	0.1	7	2	70	0	0	0	0
14	50	0.1	7	2	70	0	0	0	0
15	100	0.1	7	1	50	4.72	14.91	2.78 × 10^4^	86.85
16	100	0.1	7	1	50	2.17	16.4	3.33 × 10^4^	86.1

**Table 4:** ANOVA analysis of DTS.

Source	Sum of Squares	Mean Square	*F* Value	*p* Value
A	76.69	76.69	73.23	<0.0001
B	5.07	5.07	4.84	0.0553
C	51.44	51.44	49.12	<0.0001
D	31.73	31.73	30.29	0.0004
E	4.98	4.98	4.76	0.0570
BE	27.75	27.75	26.49	0.0006
R^2^ = 95.45% Adjusted R^2^ = 92.41%				

A: The ratio of water-based light-cured PU in the PU/TPU mixture (%) B: The HEMA addition amount (the times the weight of the PU/TPU mixture) C: Curing time per layer (s) D: Pore size of the scaffold (mm) E: Porosity of the scaffold (%).

**Table 5:** ANOVA analysis of Young’s modulus.

Source	Sum of Squares	Mean Square	*F* Value	*p* Value
A	1602.20	1602.20	276.78	<0.0001
B	22.21	22.21	3.84	0.0786
C	402.30	402.30	69.50	<0.0001
E	3.02	3.02	0.52	0.4867
BE	136.36	136.36	23.56	0.0007
R^2^ = 97.40% Adjusted R^2^ = 96.10%				

A: The ratio of water-based light-cured PU in the PU/TPU mixture (%) B: The HEMA addition amount (the times the weight of the PU/TPU mixture) C: Curing time per layer (s) D: Pore size of the scaffold (mm) E: Porosity of the scaffold (%).

**Table 6:** ANOVA analysis of the cell adhesion amount.

Source	Sum of Squares	Mean Square	*F* Value	*p* Value
A	100.53	100.53	63.08	<0.0001
B	3.65	3.65	2.29	0.1645
C	25.53	25.53	16.02	0.0031
D	58.92	58.92	36.96	0.0002
E	9.81	9.81	6.61	0.0349
BC	13.59	13.59	8.53	0.0170
R^2^ = 93.66% Adjusted R^2^ = 89.44%				

A: The ratio of water-based light-cured PU in the PU/TPU mixture (%) B: The HEMA addition amount (the times the weight of the PU/TPU mixture) C: Curing time per layer (s) D: Pore size of the scaffold (mm) E: Porosity of the scaffold (%).

**Table 7:** ANOVA analysis of the cell viability.

Source	Sum of Squares	Mean Square	*F* Value	*p* Value
A	1032.53	10,320.53	297.77	<0.0001
B	3515.90	3515.90	101.44	<0.0001
C	10,783.78	10,783.78	311.14	<0.0001
D	1378.64	1378.64	39.78	0.0001
E	17,006.77	17,006.77	490.68	<0.0001
BE	480.49	480.49	13.86	0.0047
R^2^ = 99.29% Adjusted R^2^ = 98.81%				

A: The ratio of water-based light-cured PU in the PU/TPU mixture (%) B: The HEMA addition amount (the times the weight of the PU/TPU mixture) C: Curing time per layer (s) D: Pore size of the scaffold (mm) E: Porosity of the scaffold (%).

**Table 8:** The optimal factor-level combination.

Run #	A	B	C	D	E	DTS (MPa)	Young’s Modulus (MPa)	Cell Adhesion Amount (pc)	Cell Miability (%)
1	100	0.17	13.2	1.96	50	2.98	21.42	2.43 × 10^4^	136.64
2	81.8	0.25	17.98	1.94	50	3.00	20.06	2.11 × 10^4^	147.67
3	100	0.28	7.21	1.82	50	3.00	22.44	2.08 × 10^4^	138.22
4	76.4	0.3	23.08	2.00	60	2.91	20.00	1.98 × 10^4^	125.94

A: The ratio of water-based light-cured PU in the PU/TPU mixture (%) B: The HEMA addition amount (the times the weight of the PU/TPU mixture) C: Curing time per layer (s) D: Pore size of the scaffold (mm) E: Porosity of the scaffold (%).

**Table 9:** Optimization parameter verification.

	A	B	C	D	E	DTS (MPa)	Young’s Modulus (MPa)	Cell Adhesion Amount (pc)	Cell Viability (%)
Expectation value	76.4	0.3	23.08	2	60	2.91	20.00	1.98 × 10^4^	125.94
Experimental value	76.4	0.3	23	2	60	3.05	22.37	2.75 × 10^4^	106.89
Deviation (%)	0	0	−0.35	0	0	4.59	+10.59	+28	−17.82

A: The ratio of water-based light-cured PU in the PU/TPU mixture (%) B: The HEMA addition amount (the times the weight of the PU/TPU mixture) C: Curing time per layer (s) D: Pore size of the scaffold (mm) E: Porosity of the scaffold (%).

## Data Availability

Data is contained within the article.

## References

[1] Shimomura K., Moriguchi Y., Murawski C.D., Yoshikawa H., Nakamura N. (2014). Osteochondral tissue engineering with biphasic scaffold: Current strategies and techniques. Tissue Eng. Part B Rev..

[2] Vinatier C., Mrugala D., Jorgensen C., Guicheux J., Noel D. (2009). Cartilage engineering: A crucial combination of cells, biomaterials and biofactors. Trends Biotechnol..

[3] Gillogly S.D., Voight M., Blackburn T. (1998). Treatment of articular cartilage defects of the knee with autologous chondrocyte implantation. J. Orthop. Sports Phys. Ther..

[4] Newman A.P. (1998). Articular cartilage repair. Am. J. Sports Med..

[5] Santo V.E., Gomes M.E., Mano J.F., Reis R.L. (2013). Controlled release strategies for bone, cartilage, and osteochondral engineering—Part I: Recapitulation of native tissue healing and variables for the design of delivery systems. Tissue Eng. Part B Rev..

[6] Ochi M., Uchio Y., Kawasaki K., Wakitani S., Iwasa J. (2002). Transplantation of cartilage-like tissue made by tissue engineering in the treatment of cartilage defects of the knee. J. Bone Jt. Surg. Br..

[7] Peterson L., Brittberg M., Kiviranta I., Akerlund E.L., Lindahl A. (2002). Autologous chondrocyte transplantation. Biomechanics and long-term durability. Am. J. Sports Med..

[8] Yates J.W.J. (2003). The effectiveness of autologous chondrocyte implantation for treatment of full-thickness articular cartilage lesions in workers’ compensation patients. Orthopedics.

[9] Kalson N.S., Gikas P.D., Briggs T.W.R. (2010). Current strategies for knee cartilage repair. Int. J. Clin. Pract..

[10] Huneault M.A. (2006). Preparation of interconnected poly (ε-caprolactone) porous scaffolds by a combination of polymer and salt particulate leaching. Polymer.

[11] Rakovsky A., Gotman I., Rabkin E., Gutmanas E.Y. (2014). β-TCP-polylactide composite scaffolds with high strength and enhanced permeability prepared by a modified salt leaching method. J. Mech. Behav. Biomed. Mater..

[12] Hutmacher D.W. (2001). Scaffold design and fabrication technologies for engineering tissues-state of the art and future perspectives. J. Biomater. Sci. Polym. Ed..

[13] Sun B., Long Y.Z., Zhang H.D., Li M.M., Duvail J.L., Jiang X.Y., Yin H.L. (2014). Advances in three-dimensional nanofibrous macrostructures via electrospinning. Prog. Polym. Sci..

[14] Pang L., Hao W., Jiang M., Huang J., Yan Y., Hu Y. (2013). Bony defect repair in rabbit using hybrid rapid prototyping polylactic-co-glycolic acid/beta-tricalciumphosphate collagen I/apatite scaffold and bone marrow mesenchymal stem cells. Indian J. Orthop..

[15] Park S.H., Park D.S., Shin J.W., Kang Y.G., Kim H.K., Yoon T.R., Shin J.-W. (2012). Scaffolds for bone tissue engineering fabricated from two different materials by the rapid prototyping technique: PCL versus PLGA. J. Mater. Sci. Mater. Med..

[16] Jiang C.-P., Chen Y.-Y., Hsieh M.-F. (2013). Biofabrication and in vitro study of hydroxyapatite/mPEG-PCL-mPEG scaffolds for bone tissue engineering using air pressure-aided deposition technology. Mater. Sci. Eng. C Mater. Biol. Appl..

[17] Xu T., Binder K.W., Albanna M.Z., Dice D., Zhao W., Yoo J.J., Atala A. (2013). Hybrid printing of mechanically and biologically improved constructs for cartilage tissue engineering applications. Biofabrication.

[18] Hung K.-C., Tseng C.-S., Hsu S. (2014). Synthesis and 3D printing of biodegradable polyurethane elastomer by a water-based process for cartilage tissue engineering applications. Adv. Healthc. Mater..

[19] Ding C., Qiao Z., Jiang W., Li H., Wei J., Zhou G., Dai K. (2013). Regeneration of a goat femoral head using a tissue-specific, biphasic scaffold fabricated with CAD/CAM technology. Biomaterials.

[20] Yeong W., Sudarmadji N., Yu H., Chua C., Leong K., Venkatraman S. (2010). Porous polycaprolactone scaffold for cardiac tissue engineering fabricated by selective laser sintering. Acta Biomater..

[21] Tsai K.-Y., Lin H.-Y., Chen Y.-W., Lin C.-Y., Hsu T.-T., Kao C.-T. (2017). Laser sintered magnesium-calcium silicate/poly-ε-caprolactone scaffold for bone tissue engineering. Materials.

[22] Gauvin R., Chen Y.C., Lee J.W., Soman P., Zorlutuna P., Nichol J.W., Bae H., Chen S., Khademhosseini A. (2012). Microfabrication of complex porous tissue engineering scaffolds using 3D projection stereolithography. Biomaterials.

[23] Dean D., Jonathan W., Siblani A., Wang M.O., Kim K., Mikos A.G., Fisher J.P. (2012). Continuous digital light processing (cDLP): Highly accurate additive manufacturing of tissue engineered bone scaffolds. Virtual Phys. Prototyp..

[24] Choi J.-W., Wicker R., Lee S.-H., Choi K.-H., Ha C.-S., Chung I. (2009). Fabrication of 3D biocompatible/biodegradable micro-scaffolds using dynamic mask projection microstereolithography. J. Mater. Process. Technol..

[25] Chia H.N., Wu B.M. (2015). Recent advances in 3D printing of biomaterials. J. Biol. Eng..

[26] Elomaa L., Teixeira S., Hakala R., Korhonen H., Grijpma D.W., Seppälä J.V. (2011). Preparation of poly (ε-caprolactone)-based tissue engineering scaffolds by stereolithography. Acta Biomater..

[27] Melchels F.P., Feijen J., Grijpma D.W. (2009). A poly (D, L-lactide) resin for the preparation of tissue engineering scaffolds by stereolithography. Biomaterials.

[28] Seck T.M., Melchels F.P., Feijen J., Grijpma D.W. (2010). Designed biodegradable hydrogel structures prepared by stereolithography using poly (ethylene glycol)/poly (d, l-lactide)-based resins. J. Control Release.

[29] Bens A., Seitz H., Bermes G., Emons M., Pansky A., Roitzheim B., Tobiasch E., Tille C. (2007). Non-toxic flexible photopolymers for medical stereolithography technology. Rapid Prototyp. J..

[30] Sharifi S., Mirzadeh H., Imani M., Atai M., Ziaee F. (2008). Photopolymerization and shrinkage kinetics of in situ crosslinkable N-vinyl-pyrrolidone/poly (ε-caprolactone fumarate) networks. J. Biomed. Mater. Res. Part A.

[31] Melchels F.P.W., Bertoldi K., Gabbrielli R., Velders A.H., Feijen J., Grijpma D.W. (2010). Mathematically defined tissue engineering scaffold architectures prepared by stereolithography. Biomaterials.

[32] Han L.-H., Mapili G., Chen S., Roy K. (2008). Projection microfabrication of three-dimensional scaffolds for tissue engineering. J. Manuf. Sci. Eng..

[33] Tanaka Y., Yamaoka H., Nishizawa S., Nagata S., Ogasawara T., Asawa Y., Fujihara Y., Takato T., Hoshi K. (2010). The optimization of porous polymeric scaffolds for chondrocyte/atelocollagen based tissue-engineered cartilage. Biomaterials.

[34] Shie M.-Y., Chang W.-C., Wei L.-J., Huang Y.-H., Chen C.-H., Shih C.-T., Chen Y.-W., Shen Y.-F. (2017). 3D printing of cytocompatible water-based light-cured polyurethane with hyaluronic acid for cartilage tissue engineering applications. Materials.

[35] Avasatthi V., Pawar H., Dora C.P., Bansod P., Gill M.S., Suresh S. (2016). A novel nanogel formulation of methotrexate for topical treatment of psoriasis: Optimization, in vitro and in vivo evaluation. Pharm. Dev. Technol..

[36] Schuurman W., Levett P.A., Pot M.W., van Weeren P.R., Dhert W.J.A., Hutmacher D.W., Melchels F.P.W., Klein T.J., Malda J. (2013). Gelatin-methacrylamide hydrogels as potential biomaterials for fabrication of tissue-engineered cartilage constructs. Macromol. Biosci..

[37] Wakitani S., Kimura T., Hirooka A., Ochi T., Yoneda M., Yasui N., Owaki H., Ono K. (1989). Repair of rabbit articular surfaces with allograft chondrocytes embedded in collagen gel. J. Bone Jt. Surg. Br..

[38] Triaminingsih S., Eriwati Y.K., Harahap S.A., Agustina R.G. (2018). Influence of curing time and color shade on diametral tensile strength of bulk fill composite resins. J. Int. Dent. Med. Res..

[39] (2009). ISO 10993-5:2009 Biological Evaluation of Medical Devices. Part 5: Tests for In Vitro Cytotoxicity.

[40] Chiu Y.-C., Shen Y.-F., Lee A.K.-X., Lin S.-H., Wu Y.-C., Chen Y.W. (2019). 3D printing of amino resin-based photosensitive materials on multi-parameter optimization design for vascular engineering applications. Polymers.

[41] Woodfield T.B.F., Malda J., de Wijn J., Peters F., Riesle J., van Blitterswijk C.A. (2004). Design of porous scaffolds for cartilage tissue engineering using a three-dimensional fiber-deposition technique. Biomaterials.

[42] Dai W., Kawazoe N., Lin X., Dong J., Chen G. (2010). The influence of structural design of PLGA/collagen hybrid scaffolds in cartilage tissue engineering. Biomaterials.

[43] Li L., Yu F., Shi J., Shen S., Teng H., Yang J., Wang X., Jiang Q. (2017). In situ repair of bone and cartilage defects using 3D scanning and 3D printing. Sci. Rep..

